# Balance ability and postural stability among patients with painful shoulder disorders and healthy controls

**DOI:** 10.1186/1471-2474-14-282

**Published:** 2013-10-02

**Authors:** Tobias Baierle, Thilo Kromer, Carmen Petermann, Petra Magosch, Hannu Luomajoki

**Affiliations:** 1 Institute of Physiotherapy, ATOS-Clinic, Luisenstrasse 14, Heidelberg 69115, Germany; 2 Department of Physiotherapy, SRH University Heidelberg, School of Therapeutic Sciences, Maassstrasse 26, Heidelberg 69123, Germany; 3 Department of Surgery, Sports Medicine and Medical Information Technology, ATOS-Clinic, Bismarckstrasse 9-15, Heidelberg 69115, Germany; 4 ZHAW Zurich University of Applied Sciences, Technikumstrasse 71, Winterthur 8401, Switzerland

**Keywords:** Balance ability, Pain, Postural stability, Sensorimotor system, Shoulder

## Abstract

**Background:**

In therapeutic settings, patients with shoulder pain often exhibit deficient coordinative abilities in their trunk and lower extremities. The aim of the study was to investigate 1) if there is a connection between shoulder pain and deficits in balance ability and postural stability, 2) if pain intensity is related to balance ability and postural stability, and 3) if there is a connection between body mass index (BMI) and balance ability and postural stability.

**Methods:**

In this case–control study, patients (n = 40) with pathological shoulder pain (> 4 months) were matched with a healthy controls (n = 40) and were compared with regard to their balance ability and postural stability. Outcome parameters were postural stability, balance ability and symmetry index which were measured using the S3-Check system. In addition, the influence of shoulder pain intensity and BMI on the outcome parameters was analysed.

**Results:**

Patients with shoulder pain showed significantly worse results in measurements of postural stability right/left (p < 0.01) and front/back (p < 0.01) as well as balance ability right/left (p = 0.01) and front/back (p < 0.01) compared to healthy controls. There were no significant group differences with regard to symmetry index. However, there was a significant (p < 0.01) symmetry shift towards the affected side within the shoulder pain group. There was no correlation between pain intensity and measurements of balance ability or postural stability. Likewise, no correlation between BMI and deficiencies in balance ability and postural stability was established.

**Conclusions:**

Patients with pathological shoulder pain (> 4 months) have deficiencies in balance ability and postural stability; however the underlying mechanisms for this remain unclear. Neither pain intensity nor BMI influenced the outcome parameters. Patients with shoulder pain shift their weight to the affected side. Further research is needed to determine if balance training can improve rehabilitation results in patients with shoulder pathologies.

## Background

Unrestricted shoulder function is largely dependent on the stability of the trunk, which in turn, is closely linked to the stability of the lower extremities and to balance control. The shoulder girdle has to compensate a loss of core stability and/or a deficient coordination of legs, torso or scapula by increasing movement speed and/or strength. For patients with shoulder pathologies, it is unclear whether the deficits in balance ability contribute to or is rather a consequence of the shoulder pathology.

Current evidence shows that patients with shoulder pain often suffer from proprioceptive deficits in the shoulder [1], as well as coordination deficits in their trunk and lower extremities [2-4]. As Myers et. al explain, these proprioceptive deficits lead to abnormal proprioception within the entire muscle chain, which ultimately affects central control [1]. Thus, somatosensory deficiencies in one area of the body, such as the lower extremities or the trunk, can lead to general functional problems in the shoulder area [3].

Treede et. al suggest that pain processing may cause balance disorders [5]. One explanation for this could be the fact that pain processing, the balance control circuit [5] and the inhibition of muscles caused by pain share some pathways of the central nervous system [6]. Not only pain in the spine, but also pain in the lower extremities might have a negative influence on motor control through inhibition of the musculature and/or changes of the proprioceptive feedback in painful structures [6,7]. According to Sibley et. al, pain causes presynaptic inhibition of muscle afferents [8]. Carpa and Ro showed that pain in the masticatory muscles can alter the central modulation, which in turn, influences the proprioceptive muscle spindles [9]. These muscular inhibition mechanisms due to pain might have a negative effect on balance ability.

Similarly, pain could have an effect on balance by disrupting neural speed processing. Research conducted by Luoto et. al showed that the speed of central information processing is decelerated when patients suffer from pain in the lumbar spine [10]. It remains unclear as to whether pain from shoulder pathologies could also result in a deficient ability to balance either by 1) disrupting the central information processing speed, or by 2) inhibiting the musculature or proprioception.

Other factors known to influence balance are cognitive process, such as attention [11,12] and characteristics such as age [13,14], sex and Body Mass Index (BMI) [15,16].

To our knowledge, no studies have examined the influence of pathological shoulder pain on the balance system. The aim of this study was to investigate 1) if there is a connection between shoulder pain and deficits in balance ability and postural stability, 2) if pain intensity is related to balance ability and postural stability, and 3) if there is a connection between body mass index and balance ability and postural stability. Patients with pathological shoulder pain were compared to a healthy control group in regards to balance ability, postural stability (stability index, sensorimotor index and symmetry index) and body symmetry using the S3-Check system (MFT - Multifunktionale Trainingsgeräte GmbH, Grosshoeflein, Germany).

## Methods

### Subjects

**Figure 1:**
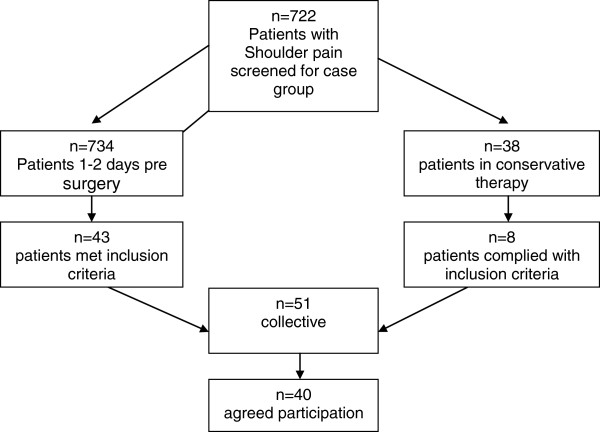
**Flow of recruitment for the case group.** Note: n = number of participants.

Subjects were recruited from the ATOS-Clinic Heidelberg, Germany which specialises in shoulder surgery and an affiliated rehabilitation centre. Forty subjects with a mean age of 55.9 years, standard deviation (SD ± 11.1) were enrolled in the patient group. The control group (n = 40) with a mean age (SD) of 56.7 (± 12.5) years, were primarily administrative staff from the ATOS-Clinic and the rehabilitation centre or were family members of patients. Written informed consent was obtained from all study participants. Inclusion criteria for the patient group were 1) pain originating in the shoulder, with a concurrent diagnosis by a physician specialised in shoulders, 2) musculoskeletal shoulder pain for more than 4 months with a pain intensity rating of at least 7 on a 15-point visual analogue scale (VAS) within the past 7 days of screening, 3) complaints of shoulder pain at rest during the screening examination. Exclusion criteria for both the patient group and the control group were 1) history of major surgery on the lower extremities (e.g. knee prosthesis or cruciate ligament plastics), 2) an injury of the lower extremities during the last six months which affected functional capabilities, 3) acute or chronic pain (including muscular pain) in the spine or lower extremities, 4) any kind of neurological complaint, 5) chronic headache, Morbus Mènière and diseases of the inner ear, 6) cardiovascular diseases which affect balance, 7) acute dizziness, 8) depression, 9) current intake of medication affecting the central nervous system (e.g. opiates), 10) cardiovascular medications which could cause balance issues, and 11) subjects who performed balance training (to avoid measurement bias). Patients (n = 734) who came to the clinic for a preliminary consultation with their treating physician regarding an upcoming shoulder surgery filled out an initial screening questionnaire. The questionnaires were reviewed and potential participants were evaluated by three trained examiners. Of those screened, 43 patients met the inclusion criteria and 33 were willing to participate. In addition, patients (n = 38) were recruited (same process as above) from the affiliated rehabilitation centre where they had an initial examination due to chronic shoulder pain. Eight met the inclusion criteria and 7 were willing to participate. From a total of 772 patients who were screened for participation, 51 met the inclusion criteria. Eleven patients declined due to lack of time, leaving 40 subjects (aged 35–80) that consented and were enrolled in the patient group for the study (Figure 1). All procedures were approved by the local ethics committee of the ATOS-Clinic Heidelberg, Germany.

### Experimental design

This case–control study was conducted at the ATOS-Clinic Heidelberg, Germany from January until June, 2011. The study examined balance ability, postural stability and body symmetry of subjects with shoulder pain for more than 4 months with a concurrent pathological shoulder diagnosis. For each patient selected, one healthy control was matched according to age (in decade units) and sex.

### Parameters

Anthropometric data- age, sex, body height, body weight- and pain parameters were collected during the screening process (Table 1). BMI was calculated by the study examiners based on the height and weight. The specific pain parameters included the following: pain duration (> 4 months or > 6 months), pain characteristics (intermittent/constant, pain at rest) and pain intensity (VAS 0–15, 0 = free of pain, 15 = maximal pain). The 15-point VAS scale was chosen in order to be consistent with the 15-point pain scale used in the Constant Score (shoulder assessment), which was familiar to the patients. The type of shoulder pathology (Table 2) and secondary diagnoses, including previous operations and medications were also collected. The following data, which are measures of the body’s stability, were collected using the S3-Check system (MFT - Multifunktionale Trainingsgeräte GmbH, Grosshoeflein, Germany)

**Table 1:** Anthropometric data of subjects

	**Control group (n = 40)**	**Shoulder pathology (n = 40)**	**P-value**
Age (years)	56.7 ± 12.5	55.9 ± 11.1	0.62
Age range	31-80	35-80	
Women (in%)	16 (40)	16 (40)	
Men (in%)	24 (60)	24 (60)	
Body weight (kg)	75.7 ± 11.9	84.5 ± 16.1	0.00
Body height (cm)	173.6 ±10.1	175.18 ± 9.9	0.22
BMI	25.1 ± 3.8	27.4 ± 3.9	0.00
BMI range	18.3 - 38.4	18.6 - 36.8	
Affected shoulder (dominant side in%)		52	
Overall mean of pain 0/15 VAS		9.6 ± 2.0	
Pain range 7–11 VAS		33	
Pain range 12–15 VAS		7	
Pain duration > 4		8	
Pain duration > 6 months		32	

Values presented as mean ± Standard Deviation (SD). p < 0.05.

**Table 2:** Distribution of the shoulder pathologies

	**Shoulder pathologies (n = 40)**
Frozen shoulder	6
Arthrosis acromio-clavicular joint and/or the humero-scapular joint	8
Tendon rupture	14
Impingement	7
Instability	2
Stable fracture e.g. before implant removal	3

• the stability index right/left using the postural stability scale,

• the stability index front/back using the postural stability scale,

• the sensorimotor index right/left using the balance ability scale,

• the sensorimotor index front/back using the balance ability scale,

• the symmetry index right/left using the symmetry scale,

• the symmetry index right/left using the symmetry scale.

### Measurement system S3-check

The S3-Check (MFT - Multifunktionale Trainingsgeräte GmbH, Grosshoeflein, Germany) is a testing system for determining the balance ability and postural stability as well as the sensorimotor regulation ability. A sensor which collects the data using specialised computer software (BITsoft, Grosshoefflein, Germany) is integrated into a uniaxial balance plate [17]. The plate can be tilted by up to 12°. Tilting motions of the measuring plate are transmitted to an inclination sensor. The measuring zone ranges from +20° to −20° with a measuring accuracy of 0.5°. The sampling rate is 100 Hz. Testing directions can be set to “front/back” and “right/left”. The system measures the magnitude and number of movements. These two measurements comprise the sensorimotor index. The symmetry index is calculated by assessing the deviation from the center of the plate (optimal value 50% right to 50% left and 50% front to 50% back, respectively). The stability index is a calculation based on the sensorimotor index and the symmetry index. Collectively, the results of these parameters provide a quantitative measure of the subject’s complex sensorimotor skills, balance ability and postural stability [18].

### Measurement procedure using the S3-system

**Figure 2:**
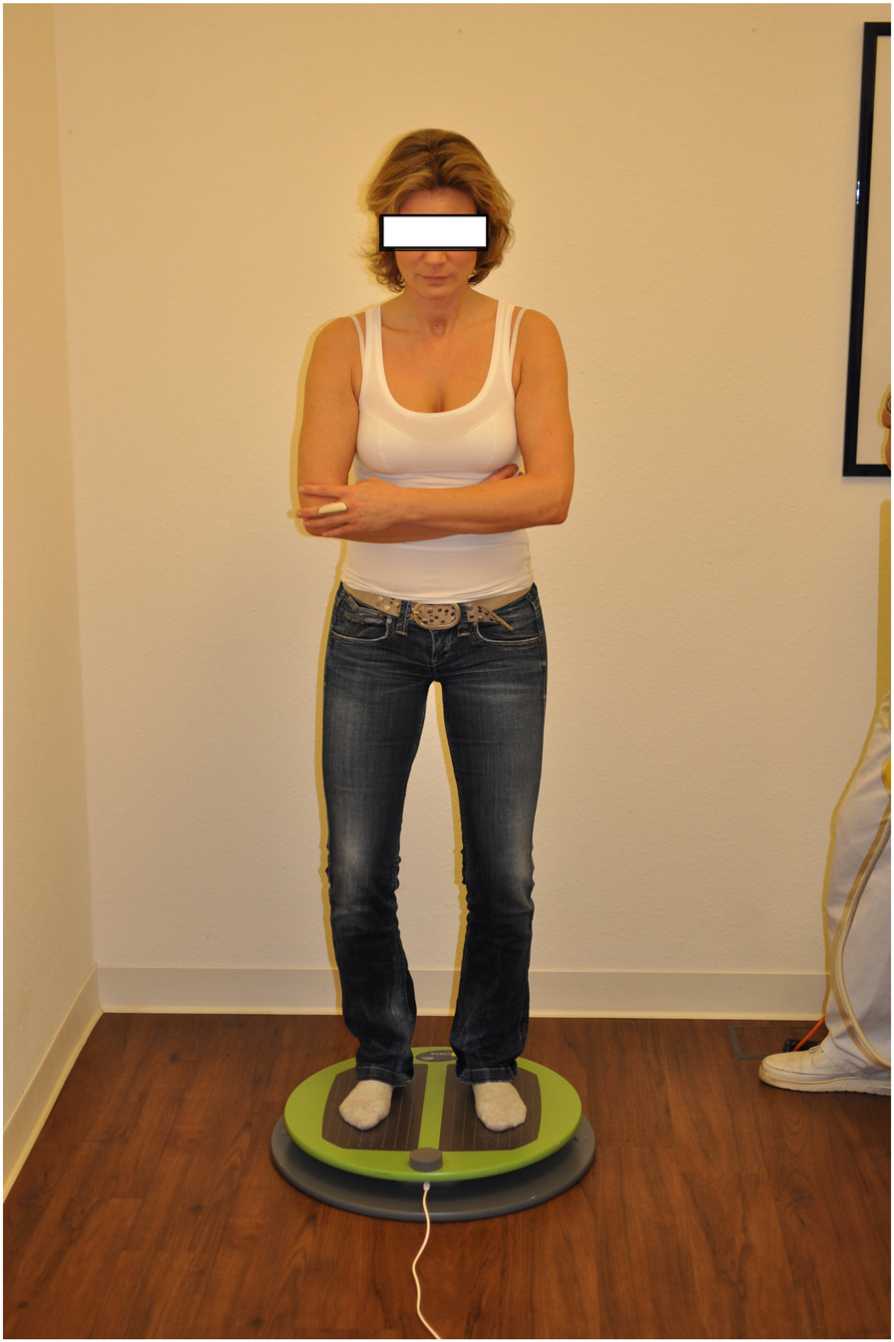
**Posture while using S3-check.** Note: Subject on balance board of S3-Check for right/left-measurement. Tests were performed with crossed arms to eliminate compensatory arm movements.

The testing was performed in a calm environment. Study subjects were instructed to remove their shoes and position their feet in a bipedal stance on the balance plate. Tests were performed with arms crossed in front of the body to eliminate compensatory arm movements during the tests and to minimize the influence of shoulder dysfunction (Figure 2). During a warm-up phase (30 second exercise simulating the actual test), the subject familiarised himself with the equipment. An attendant was placed close to the patient during testing in order to minimise the fear of falling, which could modify the outcome measures. The test itself consisted of two randomised consecutive measurement series for each testing direction:

• “front/back”

• “right/left”

The subject had to hold the balance plate in a horizontal position without receiving visual feedback from a monitor. There was a 30-second break in between both testing directions. The best test from the two series of the respective testing direction was used for further analysis.

### Quality criteria of the S3-check system

An evaluation of the interrater-reliability [17] of the S3-Check system (MFT - Multifunktionale Trainingsgeräte GmbH, Grosshoeflein, Germany) has shown a very high (from 0.90 to 0.98) intraclass correlation coefficient (ICC) of the stability index (postural stability) “right/left” and “front/back”. The correlation of the sensorimotor index (sensorimotor regulation function) “right/left” is considered as high (ICC from 0.82 to 0.84); the correlation for “front/back” is considered as medium (ICC from 0.67 to 0.69). Also test -retest reliability has been shown to be very high for the different measures (ICC from 0.90 to 0.98) [17]. Validity testing according to Raschner et al. [17] was verified by significant differences between athletes and study subjects. By fulfilling general quality criteria, the S3-Check (MFT - Multifunktionale Trainingsgeräte GmbH, Grosshoeflein, Germany) [19,20] qualifies as a suitable measuring system.

### Statistical analysis

The statistical power was calculated using G Power 3.1.2 (Faul, Universität 2009, Kiel, Germany). Statistical analysis was performed by SPSS version 19 (IBM® SPSS Statistics 19, IBM GmbH, München, Germany).

Statistical significance level was set at the p < 0.05. Normal distribution was examined using the Kolmogorov-Smirnov-Test. Univariate analysis of an association between shoulder pain, shoulder pathology, pain intensity, balance ability and postural stability was performed using Spearman’s rho correlation. To detect differences of parameters within each group of patients the Wilcoxon signed rank sum test and to detect differences of parameters between both groups the Mann–Whitney-U-Test was used. The chi-square-test measured the relative frequency which shows if there is a shift tendency of symmetry to or away from the pathological shoulder.

## Results

### Subjects

There were no significant differences between the control group (n = 40) and the subject group (n = 40) regarding age (56.7 ± 12.5 years vs. 55.9 ± 11.1; p = 0.62) and sex (40% women). The study subjects’ average pain intensity was 9.6 (± 2.0), assessed by a 15-point VAS with the majority of the subjects (n = 33) reporting pain levels ranging from 7-11/15 VAS. The number of patients whose pain duration was stated as > 4 months or > 6 months was 8 and 32, respectively. The BMI of the patient group was significantly higher (25.1 ± 3.8 vs. 27.4 ± 3.9; p < 0.01) than the BMI of the control group (Table 1). The most common shoulder pathology was a tendon rupture. In 52% of subjects the affected side was the dominant arm (Table 2).

### Relationship between shoulder pain and disorders of balance ability and postural stability

**Figure 3:**
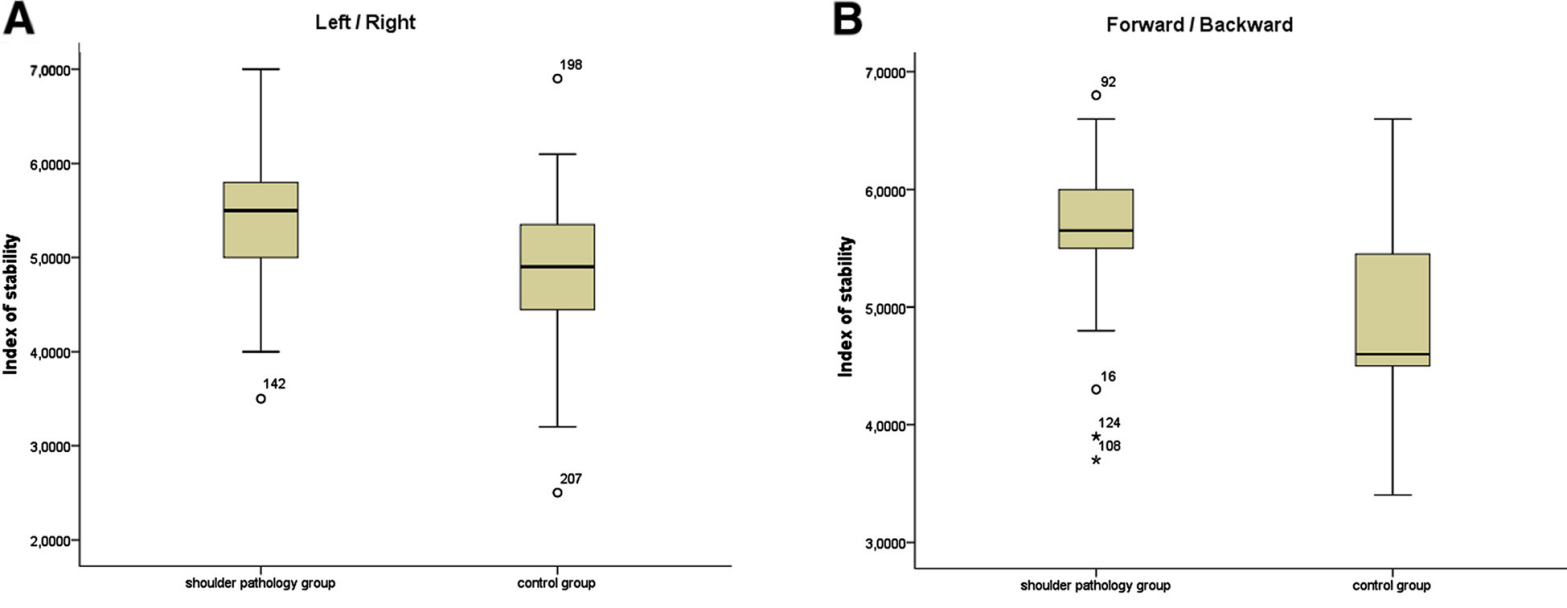
**Comparison of measurements of the stability index of both groups.** Note: Comparison of the stability index left/right and front/back of shoulder group and control group.

**Figure 4:**
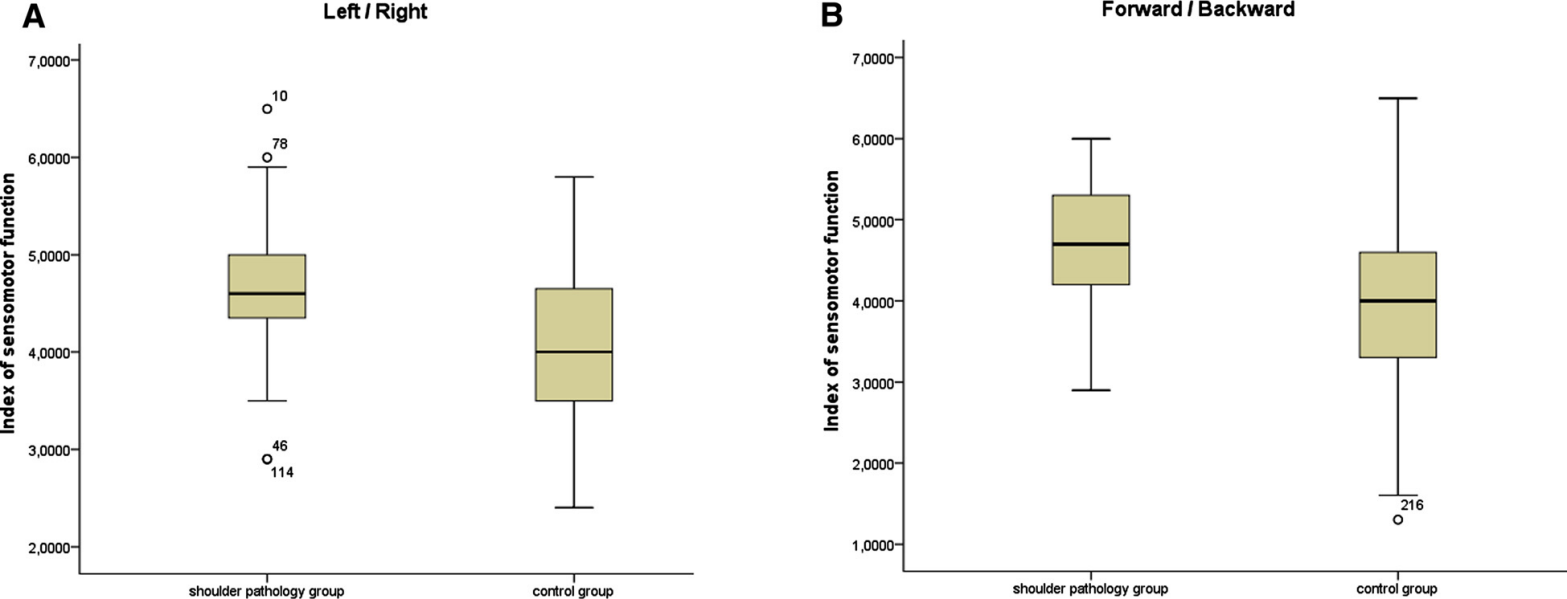
**Comparison of measurements of the sensorimotor index of both groups.** Note: Comparison of sensorimotor index left/right and front/back of shoulder pain group and control group.

When comparing the control group and the shoulder pain group (Figures 3 and 4) regarding all six measured parameters of body stability, the control group generally showed lower, or significantly lower, values than the shoulder pain group (stability index right/left p = 0.01, stability index front/back p < 0.01, sensorimotor index right/left p = 0.01, sensorimotor index front/back p = 0.01, symmetry index right/left p = 0.69 and symmetry index front/back p = 0.96). In the analysis of individual values of the symmetry index right/left of subjects with shoulder pathology, significant results (p < 0.01) were found. Twenty-two subjects (55%) showed a symmetry shift towards the affected side, 16 subjects (40%) showed a shift away from the affected side and 2 (5%) subjects had ideal symmetry index values. These results demonstrate a possible association between shoulder pain and body symmetry disorders.

In addition, the effect size and the confidence intervals of both groups were calculated and can be considered as high (> 0.8) (Table 3). The post-hoc-analysis of the power calculation produces values of 0.99/0.75/0.56 for right/left and 0.98/0.99/0.05 for front/back for the stability index/sensorimotor index/symmetry index as well as an effect size of 1.27/0.71/0.49 for right/left and 0.96/1.09/0.05 for front/back for a group size of n = 40 per group. The post-hoc-analysis of the power calculation produced favourable values except for the symmetry index front/back (Table 3).

**Table 3:** Group comparison regarding confidence interval and effect size of the parameters balance ability and postural stability

	**Control group**	**Shoulder pathology**	**Effect size**
	**95% CI**	**95% CI**	
Stability index right/left	4.6-5.1	5.4-5.8	1.27
Stability index front/back	4.6-5.1	5.2-5.7	0.97
Sensorimotor index right/left	3.5-4.2	4.3-4.8	0.7
Sensorimotor index front/back	3.8-4.4	4.4-4.9	1.09
Symmetry index right/left	41.9-49.3	42.9-49.7	0.49
Symmetry index front/back	45.3-50.7	49.4-55.2	0.05

### Relationship between pain intensity and disorders of balance ability and postural stability

There was no correlation between stability index and pain intensity, and also no correlation between sensorimotor index and pain intensity (Table 4).

**Table 4:** Results of the correlation of pain intensity with balance ability and postural stability

	**Spearman**	**P value**
Stability index right/left	0.15	0.36
Stability index front/back	0.11	0.48
Sensorimotor index right/left	0.19	0.24
Sensorimotor index front/back	0.13	0.44
Symmetry index right/left	0.22	0.18
Symmetry index front/back	0.15	0.36

### Relationship between BMI and a body stability

Neither the shoulder pain group nor control group showed a correlation between BMI and the balance measurements: stability index, sensorimotor index and symmetry index (Tables 5 and 6). Therefore, no correlation between BMI and deficiencies in balance ability and postural stability was established.

**Table 5:** Results of the correlation of body mass index with balance ability and postural stability in the shoulder pathology group

	**Spearman**	**P value**
Stability index right/left	0.28	0.08
Stability index front/back	0.26	0.12
Sensorimotor index right/left	0.13	0.40
Sensorimotor index front/back	0.18	0.26
Symmetry index right/left	0.20	0.21
Symmetry index front/back	0.14	0.39

Values presented as mean ± Standard Deviation (SD). p < 0.05.

**Table 6:** Correlation of body mass index with balance ability and postural stability in the control group

	**Spearman**	**P value**
Stability index right/left	0.24	0.13
Stability index front/back	0.20	0.21
Sensorimotor index right/left	0.27	0.09
Sensorimotor index front/back	0.17	0.29
Symmetry index right/left	−0.19	0.25
Symmetry index front/back	0.20	0.21

## Discussion

The study demonstrated that painful shoulder pathologies can disturb body stability, even when the influence of arm movement is restricted. Our findings showed no evidence of a correlation between pain intensity and deficits in balance ability and postural stability, and no evidence of a correlation between BMI and balance ability and postural stability.

### Relationship between shoulder pain and body stability disorders

One explanation for the disturbances in balance observed in our patients might be related to pain processing. Both pain processing and balance control circuits partly rely on the same central nervous system pathways [5,21]. Shoulder pain might alter these pathways which, in turn, could influence balance. According to Crombez et al. [22] and Ruhe et al. [16] this can be explained by pain interference which links changes in body control to pain in the lower back.

### Relationship between pain intensity and body stability disorders

The unproven relationship between pain intensity and disturbed body stability might indicate that pain could be responsible for balance issues in general.

This assumption is supported by results from Ruhe et al. [7,23] with back pain patients and Lihavainen et al. [24] with geriatric patients suffering from chronic pain. Most subjects in Lihavainen’s [24] examinations suffered from pain in the lower body or the cervical spine. The study showed the influence of pain intensity on the degree of the body stability disorder.

This is in contrast to the research of Jones et al. [25]. They examined fibromyalgia patients with sensory and cognitive deficits. They failed to find a direct connection between either balance issues and pain intensity or balance and medication (opiates and/or benzodiazepines) or balance and muscle strength. These results might indicate that the affected structures are not only inhibited directly by pain, but that there are other mechanisms which may cause balance issues as well.

So far no study has confirmed these results for shoulder pain patients.

### Relationship between BMI and body stability disorders

Our study showed no correlation between BMI and disturbances of balance ability and postural stability. While some research supports our findings, inconsistencies throughout the literature remain [15,23,26-28].

### Strengths and limitations of the study

Due to the narrowly defined inclusion criteria and comprehensive exclusion criteria of participant selection, as well as the fact that the patient group and control group were well-matched according to age and sex, some potential confounding factors were mitigated at the start of the study.

Although our study showed that, in contrast to healthy controls, patients with painful shoulder pathologies do exhibit problems in balance ability, it does not clarify the underlying mechanisms for these problems. In addition, factors such as cognitive alterations were not explored as part of this study. Because we only included patients with pain scores greater than 7/15 VAS, our findings cannot be applied to patients with pathological shoulder pain with reported pain scores of less than 7/15 VAS. Likewise, we do not know how pain duration may affect the balance system – as one would expect in neuro-physiological changes [25] – since we only included patients who suffered from pain for > 4 months.

Another limitation was the fact that conclusions could not be drawn according to shoulder pathology sub-group, due to the small sample size and varying numbers of subjects per sub-group. Considerably more subjects suffered from medium strong pain (n = 33) than from strong pain (n = 7) (see Table 1). Thus, the study can only make limited statements about the correlation of pain intensity and disturbances in balance ability and postural stability. In addition, the majority (n = 33) of patients reported a moderate pain score of 7-11/15 VAS (see Table 1) which may explain why there was no correlation of pain intensity and disturbances in balance ability and postural stability.

Further criticism concerns the comparability with other studies on balance ability, due to limitations of the S3-Check measurement system, which does not allow for testing different sensory conditions (e.g. eyes open vs. eyes closed, stable vs. instable ground). However, the quality criteria for the S-3 Check were calculated on the basis of another system of balance testing. Thus, these results cannot be compared with those from other testing systems since the demands for the sensorimotor system are different for each testing system.

### Clinical considerations

Current practice for shoulder rehabilitation focuses on improving joint mobility, muscle strength, muscle endurance and muscle balance. Although some authors [2,4] postulate that a relationship between shoulder pathologies and balance or postural control exists, substantial evidence for these theories is still lacking. Never less, it has been suggested that shoulder pathologies are negatively impacted by a loss of stability and a coordination deficiency of the proximal body parts (legs, torso and scapula). Therefore, therapists treating patients with painful shoulder pathologies should consider evaluating the balance ability and the stability of the torso and lower extremities, in addition to conventional therapy approaches. When deficiencies are detected, proprioceptive balance training, as well as adequate pain therapy could counteract potential disturbances of cognitive processes or alterations of the central signal processing, thereby improving rehabilitation outcomes.

## Conclusions

The study shows that moderate to severely-rated painful shoulder pathologies are accompanied by deficits in balance ability and postural stability. However, it can neither show a relationship between pain intensity and balance issues nor clarify the underlying mechanism. Our study could not show a correlation between BMI and balance ability or postural stability.

Further research is necessary to clarify whether an association exists between pain intensity and balance problems in patients with shoulder problems, as well as between BMI and balance problems. Futhermore, there is a need to determine other factors which influence balance ability in persons with shoulder pathologies. Exploring the link between pain treatments, such as early pain therapy, and balance issues could also contribute to finding solutions for patients with balance deficits. In addition, further research is needed to determine if balance training can improve rehabilitation results in patients with shoulder pathologies.

## Competing interests

The authors declare that they have no competing interests.

## Authors’ contributions

TB planned the study, examined the patients and was the main author of the paper. PM did the statistical analysis of the study and was responsible for the statistical methods used in the study as well as for data interpretation. HL and TK were involved in planning the study and methodological considerations, CP participated in the content, the design and helped draft the manuscript. All authors read and approved the final manuscript.

## Pre-publication history

The pre-publication history for this paper can be accessed here:

http://www.biomedcentral.com/1471-2474/14/282/prepub
